# CD4CD8αα IELs: They Have Something to Say

**DOI:** 10.3389/fimmu.2019.02269

**Published:** 2019-10-09

**Authors:** Chao Zhou, Yuan Qiu, Hua Yang

**Affiliations:** Department of General Surgery, Xinqiao Hospital, Army Medical University, Chongqing, China

**Keywords:** CD4CD8αα intestinal intraepithelial lymphocytes (CD4CD8αα IELs), transcription factors, intestinal microbes, vitamin D receptor (VDR), inflammatory bowel disease (IBD)

## Abstract

The intraepithelial lymphocytes (IELs) that reside within the epithelium of the intestine play a critical role in maintaining the immune balance of the gut. CD4CD8αα IELs are one of the most important types of IELs, and they play an irreplaceable role in maintaining the balance of the intestinal immune system. CD4CD8αα IELs are often regarded as a special subtype of CD4^+^ IELs that can express CD8αα on their cytomembrane. Hence, CD4CD8αα IELs not only have the ability to modulate the functions of immune cells but also are regarded as cytotoxic T lymphocytes (CTLs). Transcription factors, microbes, and dietary factors have a substantial effect on the development of CD4CD8αα IELs, which make them exert immunosuppression and cytotoxicity activities. In addition, there is an intimate relationship between CD4CD8αα IELs and inflammatory bowel disease (IBD), whereas it is still unclear how CD4CD8αα IELs influence IBD. As such, this review will focus on the unparalleled differentiation of CD4CD8αα IELs and discuss how these cells might be devoted to tolerance and immunopathological responses in the intestinal tract. In addition, the role of CD4CD8αα IELs in IBD would also be discussed.

## Introduction

The intestinal tract serves as the primary site of nutrient absorption in the body and harbors large amounts of microorganisms, which establish a barrier between hostile external environments and the internal milieu. The composition of the intestinal mucosal barrier is quite intricate and endows its special structure (1). The intestinal tract is constantly exposed to food and bacteria, which shapes a delicate balance between the intestine and foreign matter that exerts protective immunity against detrimental pathogens and tolerance to food and beneficial bacteria (2, 3). To maintain the intestinal barrier homeostatic balance and the integrity of the intestine, there is an experienced and complex immune system in the intestinal mucosa. Intraepithelial lymphocytes (IELs), as an indispensable part of the intestinal immune system, play a vital role in direct contact with antigens and food in the intestinal lumen to form the front line of immune defense against invading pathogens. Interestingly, IELs have a different proportion between the small intestine and the large intestine; Qiu et al. (4) reported that IELs are present at a density of one IEL per 5–10 intestinal epithelial cells (IECs) in the small intestine and one IEL per 30–50 IECs in the large intestine. These complex environments of the intestinal tract give rise to various components and complex functions of IELs. According to their developmental origins and phenotypes, IELs can be divided into two major subsets. The first one consists of mainly CD4^+^ or CD8αβ^+^TCRαβ^+^ IELs, which are regarded as “type a” IELs or “induced” IELs (5). These cells are derived from conventional CD4 or CD8αβ T cells in the periphery that undergo final differentiation in the intestine. The other subset was previously classified as “type b” IELs or “natural IELs.” Natural IELs express TCRγδ or TCRαβ but do not express either CD4 or CD8αβ. In addition, these kinds of IELs express homodimers of CD8a (CD8aa), which is regarded as a hallmark to identify and distinguish natural IELs from induced IELs in the intestinal epithelium (6, 7). The origin and function of natural IELs have been described very comprehensively in previous reviews (8). They transplant into the intestinal tract after developing to maturity in the thymus, and they rarely have crosstalk with peripheral lymphocytes in the intestinal tract. However, the study of induced IELs is still unclear. Induced IELs have intimate contact with mesenteric lymph nodes and the systemic immune system. Therefore, the exploration of induced IELs has been a hotspot in recent years. Double-positive IELs (CD4^+^CD8αα^+^ IELs, or DP IELs), which are a kind of CD4^+^ IELs with some functions of CD8^+^ IELs, belong to the induced subset. Under physiological conditions, the number of CD4^+^CD8αα^+^ IELs is very small. However, in some special cases, especially during virus invasion and intestinal inflammation, DP T cell numbers can be significantly increased in both the intestinal epithelium and lamina propria (LP) and are named CD4CD8αα IELs and CD4CD8αα LPLs, respectively (9). Therefore, it is suggested that there is intimate crosstalk between CD4CD8αα IELs and CD4CD8αα LPLs. As a special CD4^+^ IEL subset, CD4CD8αα IELs not only can assist effector T cells and B cells exert their function but also have a cytotoxic effect. In this review, we highlight recent advances in understanding lineage commitment and the functional differentiation of CD4CD8αα IELs. Then, we explore the factors that influence the development of CD4CD8αα IELs. Finally, we discuss the protective effect of these cells in IBD and how they also accelerate intestinal damage in some pathological conditions.

## The Development of CD4CD8αα IELs (Figure 1)

There is a fierce controversy about the origin and development of IELs. IELs are traditionally thought to develop in the thymus; the evidence is that IEL numbers are drastically reduced in athymic mice compared to those in normal mice (10). After transplanting neonatal thymus to athymic mice, the number of IELs can be restored (11). However, Fichtelius et al. (12) discovered that the intestinal epithelium is also a first-level lymphoid organ and that the intestinal tract retains some vestigial function in lymphopoiesis. In other words, IELs can develop independently of the thymus. This finding indicates that the thymus and intestinal epithelium are involved in IEL development. Although most IELs originate from the thymus, there are subtle differences between induced IELs and natural IELs during the development of IELs.

**Figure 1:**
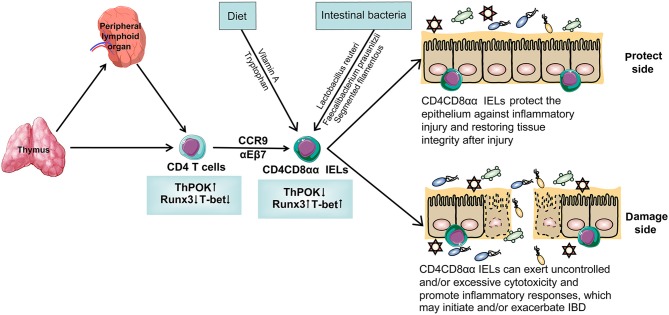
The specific differentiation and development process of CD4CD8αα IELs. T cells originate from bone marrow and most of them develop in thymus while some of them develop in peripheral lymphoid organ. T cells differentiate into CD4 T cells with the help of transcription factors that are highly expressed ThPOK and lower-expression Runx3 and T-bet. With the help of the gut-homing markers CCR9 and α4β7, CD4 T cells migrate in the intestinal epithelium and are named CD4 IELs. These cells can acquire CD8αα on their cytomembrane and become a kind of special CD4 IELs, CD4CD8αα IELs. After interacting with intestinal bacteria and diet, CD4CD8αα IELs have a different function. On the one hand, CD4CD8αα IELs protect the epithelium against inflammatory injury, restoring tissue integrity after injury. On the other hand, it can exert uncontrolled and/or excessive cytotoxicity and promote inflammatory responses, which may initiate and/or exacerbate IBD.

Natural IELs refer to the ontogeny of the precursor cells; these precursors go through an “alternative” self-antigen-based thymic maturation process in the thymus. After these cells develop into mature cells, they directly migrate to the intestinal epithelium to play a role in immunomodulation (13, 14). Moreover, the number of natural IELs will decrease with age and represent a minor IELs population at later stages in life. On the contrary, the number of induced IELs will steadily increase with age, especially when they are exposed to exogenous antigens. In addition, induced IELs derived from circulating conventional CD4 or CD8 T cells further undergo a post-thymic differentiation process. Induced IELs express TCRαβ with CD4 or CD8α and arise from conventional T cells, which are major histocompatibility complex (MHC) class II restricted and MHC class I restricted, respectively (15). These IELs are thought to have followed conventional thymic selection and reached the gut after antigenic stimulation in the periphery, particularly in gut-associated lymphoid tissue (GALT). Therefore, these kinds of IELs are incorporated into the induced IEL compartment (7). After being positively selected by the thymus and with the help of gut-homing receptors, including αEβ7 integrin and CC-chemokine receptor 9 (CCR9) and their ligand, which are produced by IECs, the mature induced IEL precursors finally migrate to the intestinal epithelium.

As special induced IELs, there are some differences between CD4CD8αα IELs and traditionally induced IELs during their development. The main difference is that they have different ways to arrive at the intestinal mucosa. Scott et al. (16) have identified that with the help of luminal bacteria, dietary constituents, and IECs, traditionally induced IELs can arrive at the intestinal tract successfully. Nevertheless, CD4CD8αα IELs still cannot arrive at the intestinal tract, and there are other local intestinal factors that take part in homing to the intestinal mucosa. Some studies have found that dendritic cells (DCs) play an important role in transmitting antigen information and that this information induces T cells to migrate from peripheral lymph nodes into the intestinal mucosa. As special DCs, CD103^+^ DCs have several unique features (17, 18). The first documented function of these cells is the capacity to imprint the expression of the gut-homing markers CCR9 and α4β7 on interacting naïve T and B cells. In addition, CD103^+^ DCs produced retinoic acid (RA) from vitamin A (retinol), which enhances the expression of α4β7 and CCR9 on T cells upon activation and imprints them with gut tropism (19). Antigen information was another important factor in inducing CD4CD8αα T cell migration to the intestine. However, antigen information still cannot successfully reach CD103^+^-LP DCs when the gut is invaded by something foreign. Because they have no ability to pass through the epithelium from the lumen, macrophages play an important role in this process; the macrophages might subsequently transfer antigens to CD103^+^-DCs in the LP. After CD103^+^-DCs received information about the pathogen, they were able to present the antigen information to CD4CD8αα T cells. Combining with the help of CCR9 and α4β7, CD4CD8αα T cells finally migrate into the intestinal epithelial from the LP and become CD4CD8αα IELs (16, 20).

## The Impact of Transcription Factors ON CD4CD8αα IELs

Much progress has been made in recent years toward understanding the development of immature T cell precursors (thymocytes) to either the CD4 or CD8 T cell lineages (21). The constitutive expression of ThPOK during thymic development leads to the redirection of class I-restricted thymocytes to the CD4 lineage (22). However, very little is known about the transcription factor network that regulates the development of CD4CD8αα IELs. Mucida et al. (23) found that CD4CD8αα IELs lack ThPOK, which is an indispensable transcription factor during the development of CD4^+^ IELs. Reis et al. (24) demonstrated that the reason why CD4CD8αα IELs lack ThPOK is that another transcription factor, Runx3, suppresses the expression of ThPOK. Additionally, they used a knock-in reporter mouse model that has a high expression of the Runx3 gene in mouse and found that the expression of CD8αα is accompanied by a high expression of Runx3. Intriguingly, CD4CD8αα IELs have a low level of ThPOK expression while the expression of Runx3 is very high. Consistent with this result, mice with a conditional deletion of Runx3 fail to repress the expression of ThPOK. Two studies independently showed that loss of ThPOK function during T cell development leads to the redirection of MHC class II-restricted cells into the CD8^+^ T cell lineage (22, 25). This result indicated that ThPOK and Runx3 are a pair of antagonistic factors during the differentiation of CD4CD8αα IELs and their cytotoxic T lymphocyte (CTL) program. Additionally, another transcription factor, T-bet, is also well-known to regulate IELs (26). On one hand, T-bet can regulate the differentiation of Th1 cells from naïve T cells and inhibit the development of other T helper subtypes. On the other hand, T-betalso plays an indispensable role in exerting the cytotoxic effect of CD8^+^ T cells. Without T-bet, initial CD8^+^ T cells cannot obtain the surface markers of effector cells under the stimulation of antigens, and their cytotoxic effect is severely impaired (27). In addition, several studies have reported that T-bet participates in regulating ThPOK-Runx3 during the differentiation process of CD4CD8αα IELs. Reis et al. (28) discovered that T-bet is an upstream regulator of ThPOK-Runx3, focusing attention on the role of the development of cytotoxic CD4CD8αα IELs. Then, the upregulation of T-bet can reduce ThPOK and induce Runx3, which promotes cytotoxic CD4CD8αα IEL differentiation. Collectively, in the lineage recommitment of the CD4CD8αα IEL process, there is a complementary relationship between T-bet and Runx3. On the one hand, they promote the differentiation of CD8^+^ T cells by activating CD8 sites; on the other hand, they silence CD4 sites and inhibit ThPOK expression. The final result is that these transcription factors are involved in conventional, MHC class II-restricted TCRαβ^+^CD4^+^ T cell differentiation into induced CD8αα^+^ IELs. However, the exact regulatory mechanisms of T-bet remain unclear. Runx–CBFβ and histone deacetylases (HDAC1 and HDAC2) may play an important role in helping T-bet regulate the differentiation of CD4CD8αα IELs. It is widely known that Runx–CBFβ complexes are essential for the development of CD8^+^ T cells and their differentiation into cytotoxic effector T cells (29). A recent study demonstrated that histone deacetylases (HDAC1 and HDAC2) maintain the integrity of the CD4 lineage. During the development of late T cells, loss of HDAC1 and HDAC2 will result in the appearance of MHC class II-selected CD4^+^ helper T cells (Th) that express CD8 lineage genes, such as Cd8a and Cd8b1. In addition, with the help of Runx–CBFβ complexes, CD4^+^ helper T cells acquire a CD8 effector program. These results demonstrate that HDAC1–HDAC2 maintains CD4 lineage integrity by repressing Runx–CBFβ complexes (30). ThPOK and Runx3 mediate the differentiation of CD4CD8αα IELs, and ThPOK is an indispensable transcription factor during the development of CD4^+^ T cells. Runx3 helps naïve T cells transform into CD8^+^ T cells. T-bet suppresses the expression of ThPOK to assist naïve T cells in transforming into CD8^+^ T cells. In contrast, HDAC1 and HDAC2 can repress Runx–CBFβ complexes to maintain CD4 lineage integrity.

Interestingly, there are also special transcription factors that are directly regulated by exogenous factors that participate in the regulation of CD4CD8αα IELs, such as the aryl hydrocarbon receptor (AHR). AHR is a ligand-dependent transcription factor; once AHR combines with its ligand, it will produce signals important in maintaining physical stability. Furthermore, an increasing number of experts suggested that AHR signaling is required for the development and maintenance of diverse IELs populations. Many previous articles have emphasized the effect of AHR on CD8^+^ IELs (31). There are shreds of evidence showing that AHR signaling is critical for the development of CD4CD8αα IELs. Here, we focus on the relationship between AHR and CD4CD8αα IELs. The differentiation of CD4CD8αα IELs from CD4^+^ IELs requires cell-intrinsic AHR activation. It is almost impossible to detect CD4CD8αα IELs in Ahr^−/−^ mice, which are AHR knockout mice and lack AHR in the body. In addition, compared with Ahr^+/+^ mice, Ahr^−/−^ mice exhibit decreased ThPOK expression. Consistent with this finding, giving mice a synthetic diet that lacks ingredients of AHR results in the same phenotype as Ahr^−/−^ mice (32). However, without AHR, CD4^+^ IELs, and TCRαβCD8αα^+^ IELs can still develop, proliferate, and home to their target organs (33). This result indicated that the reason for the decrease in CD4CD8αα IELs is that the conversion between CD4^+^ IELs and CD4CD8αα IELs is blocked. However, the conversion from CD4^+^ IELs to CD4CD8αα IELs can be successfully completed after supplying TCDD or FICZ, which are exogenous and endogenous ligands of AHR, respectively. This result suggests that the differentiation process of CD4CD8αα IELs from TCRαβ CD4^+^ IELs cannot be completed without AHR. As a nuclear transcription factor, AHR can regulate the transcription factor ThPOK-Runx3 to regulate the maturation process of CD4CD8αα IELs (34). The mutual regulation and mutually exclusive activity of these transcription factors determine the ultimate differentiation fate of naïve T cells and the formation of CD4CD8αα IELs (25).

## The Influence of Microbes and Diet on CD4CD8αα IELs

Cervantes-Barragan et al. (35) revealed that intestinal microbes are another indispensable factor during the development of precursor T cells. The interaction of the gut and microbes has a substantial influence on CD4CD8αα IELs. The taxa of microbes determine the differentiated destiny of naïve T cells. Segmented filamentous bacteria command CD4^+^ T cells to transform into Th17 cells, while Clostridia clusters IV and XIVa urge naïve T cells to become CD4^+^ T regulatory cells (Tregs) that function mainly to mediate immune tolerance. Additionally, *Faecalibacterium prausnitzii* plays an important role in the development of inducing CD4^+^ T cells that produce IL-10 to inhibit the inflammatory response (36–40). Moreover, intestinal microbes can regulate the differentiation of antigen-activated CD4^+^ T cells into CD4CD8αα IELs. Colonna et al. (41) sequenced PCR amplicons from 16S ribosomal RNA genes present in the ileum of CD4CD8αα IELs^+^ and CD4CD8αα IELs^−^ mice, which are a kind of mice in which CD4CD8αα IELs are present and absent *in vivo*, respectively. They found that there were many *Lactobacillus reuteri* bacteria in CD4CD8αα IELs^+^ mice; however, when antibiotics were administered to CD4CD8αα IELs^+^ mice, the number of *L. reuteri* quickly decreased. These results are in accordance with those of Mucida D, who reported that CD4CD8αα IELs are absent in germ-free (GF) mice. However, when GF animals are reconstituted with specific pathogen-free (SPF) microorganisms, such as *F. prausnitzii*, which is a major gut bacterium of the Clostridium IV group, the number of CD4CD8αα IELs presents an increasing trend. In addition, there is the same tendency that both the number of *F. prausnitzii* and CD4CD8αα IELs present at a decreased level in the feces of patients with IBD, indicating that some microbial factors take part directly or indirectly in the development of CD4CD8αα IELs (23, 42). However, detailed mechanisms remain unclear. In summary, intestinal microbes provide crucial signals for the development and function of CD4CD8αα IELs. However, the microbial communities and their metabolites also have an effect on the susceptibility of the host to many immune-mediated diseases and disorders when the dialog between the host and the microbes goes awry (43).

Commensal microbial mediate the maturation development of CD4CD8αα IELs by mainly decomposing the diet. Thus, there is an intriguing hypothesis that different kinds of food may also have an effect on the function of CD4CD8αα IELs. Recently, a study discovered *L. reuteri* as a commensal microbial that metabolizes tryptophan into indole derivatives, which is an activator of AHR. Colonna et al. (41) found that different concentrations of AHR ligands, such as tryptophan, have substantial effects on the number of CD4CD8αα IELs; the higher the concentration of tryptophan is, the greater the quantity of CD4CD8αα IELs in mice. Nevertheless, in GF mice, no matter how high the concentration of tryptophan, CD4CD8αα IELs still cannot be induced. Thus, intestinal microbes together with tryptophan jointly participate in the formation of CD4CD8αα IELs.

RA, the main metabolite of vitamin A, is produced by mainly GALT-resident DCs or probably by other cells, such as IECs. They have the ability to induce the expression of the gut-homing receptors α4β7-integrin and CC-chemokine receptor 9 (CCR9), which induce CD4CD8αα IELs to migrate to the intestinal epithelium. In addition, RA can antagonize the differentiation of pro-inflammatory Th17 cells by potentiating the TGFβ-mediated induction of Treg cells (44). Therefore, RA is speculated to play an indispensable role in the induction of intestinal regulatory responses (44, 45). In normal physiological conditions, RA assists GALT to generate Foxp3^+^ iTregs during the recognition of luminal antigens (46). Tregs exert a restrictive function that limits naïve T cell differentiation into effector cells so that the intestinal lumen can exist in harmony with innocuous antigens (47). Altogether, RA is a conventional protective factor. However, Depaolo et al. (48) reported that RA acted as an adjuvant that promoted rather than prevented inflammation when IL-15 and RA act at primarily the level of DCs to abrogate intestinal homeostasis. IL-15, which is expressed by the intestinal mucosa, has been shown to promote intraepithelial CTLs to become killer cells (49). Under the effect of IL-15, RA induces naïve T cells to differentiate into CD4CD8αα T cells, and the cytotoxicity of CD4CD8αα T cells disturbs the balance among the intestinal lumen and dietary antigen, innocuous commensal bacteria. Therefore, RA conventionally plays an indispensable role in maintaining immune tolerance in some special situations when combined with IL-15, and RA may be integral to IBD.

Cantorna et al. (50) revealed an intriguing discovery that people in North America and Europe have a high prevalence of IBD especially during the winter when there is a lack of sunlight, which implies that vitamin D may be an important environmental factor that is integral to IBD development. The [1,25(OH)2D3] active form of vitamin D has been regarded as an immunosuppressive agent that alleviates the pathogenesis of IBD, which stimulates mainly IELs to secrete IL-10 and TGF-β1 and inhibits inflammation in experimental mouse models of IBD (50). Yu et al. (51) discovered that the IELs of vitamin D receptor (VDR) KO mice lack the CD4CD8αα T cell population and fail to secrete IL-10, which suggests that VDR KO mice are susceptible to inflammation in the gastrointestinal tract. In addition, the lack of CD4CD8αα IELs is due in part to decreased CCR9 expression on T cells, giving rise to decreased homing of T cells to the gut. Therefore, the number of CD4CD8αα T cells is sharply reduced so that IL-10 is also lacking in the intestinal epithelium. However, expression of the VDR is important for the reacquisition of CD8 expression and homing of the largely CD4CD8αα T cells to the gut to transform into CD4CD8αα IELs, which can increase the numbers of Tregs that play a critical role in the maintenance of self-tolerance (52). Without the VDR, CD4^+^ T cells cannot acquire the expression of CD8αα and fail to home to the gastrointestinal tract, which contributes to the normal bacterial flora becoming unbalanced (51). As such, vitamin D is an important regulator of the T cell response in the intestinal lumen, which regulates the T cell response and controls immunity, inflammation, and intestinal homeostasis.

## The Function OF CD4CD8αα IELs

The special location of IELs at the intersection between the external environment and the core of the body gives them different functions. Generally, TCRγδ IELs ensure the integrity of the intestinal epithelium and maintain local immune quiescence that prevent an immune response in the gut under physiological conditions (53). CD8αα IELs have a potent antigen-dependent cytotoxic effector phenotype; they can generate granzyme and perforin to eliminate pathogens or injured epithelial cells; CD4^+^ IELs often play a helper role that assists other IELs to exert their function. However, the precise physiological functions of CD4CD8αα IELs remain unclear. Nevertheless, we can predict that these cells may play a crucial role in preventing pathogens from invading and maintaining the integrity of the epithelial barrier in the intestine. The reason we put forward this conjecture is that CD4CD8αα IELs are a special subset of CD4^+^ IELs that acquire the expression of CD8a homodimers on their cytomembrane. Therefore, they function not only as CD4^+^ IELs but also as CD8^+^ IELs. Some advanced studies have reported that the preferential loss of mucosal CD4^+^ T cells from both the LP and epithelial compartments during SIV or HIV infections impairs the integrity of the mucosal barrier and leads to translocation of enteric bacteria and increased local and systemic infections (54, 55). Similar to other subsets of IELs, CD4CD8αα IELs are “activated yet resting,” which are useless under a physiological situation (56). However, once pathogens invade, these cells are immediately activated (57). In line with this effect, Mucida et al. (23) demonstrated that antigen-specific CD4CD8αα IELs are generated by transferring OVA-specific OT-II cells into immunodeficient recipients following OVA feeding and they remain immunologically inactive. In another study, Hatano et al. (58) observed that antigen presentation by small IECs uniquely enhances IFN-γ secretion from CD4^+^ IELs and that diet can induce CD4CD8αα IELs to become immunologically active, producing a large amount of IFN-γ and TNF-α and upregulating the expression of CD107a. These findings indicate that the cells have cytotoxic potential (23). In most cases, although CD4CD8αα IELs have cytotoxic properties, they exhibit the immunologically quiescent properties. Guillaume et al. (59) described CD4CD8αα IELs reactive to the gut bacterium *F. prausnitzii* and endowed with regulatory/suppressive functions. CD4CD8αα IELs are abundant in the healthy colonic mucosa but less common in that of patients with inflammatory bowel disease (IBD). These observations further demonstrate the important contributions of CD4CD8αα IELs to the regulation of immune responses and the maintenance of immune quiescence in the intestine. It is necessary to note the plasticity of CD4CD8αα IELs in their function. Reis et al. (24) established a T cell-transfer model of colitis with the generation of CD4CD8αα IELs, along with reduced intestinal inflammation. Overall, CD4CD8αα IELs have the ability to maintain intestinal homeostasis and preclude an exaggerated response toward luminal contents, whether harmful or beneficial.

## The Roles of CD4CD8αα IELs IN IBD

IBD is regarded as chronic relapsing inflammation. Several studies have demonstrated that IBD is a T cell-mediated autoimmune disease that can occur in the small intestine, the large intestine, or both (60). IELs are perfectly positioned within the intestinal epithelium to provide the first line of mucosal defense against luminal microbes or rapidly respond to epithelial injury. Their special position predestined them to participate in the early immune reaction (61). Ooi et al. (62) believed that the cause of IBD is inappropriate T cell responses to the normal bacterial flora in the gut. Therefore, to maintain the intestinal balance of physiological conditions, some cells must play an indispensable role. Honda et al. (63) discovered that CD4CD8αα IELs play an immune suppression role that produces IL-10, inhibiting the intestinal tract from reacting with the intestinal tract microbes under physiological conditions. In addition, CD4CD8αα IELs can secrete many cytokines, such as IL-10 and TGF-β, which can prevent Th1-induced intestinal inflammation. Interestingly, when the intestine is invaded by pathogens, double-positive T cells increasingly move into the epithelial layer from the LP and secrete protective cytokines, while these cells, which are located in the LP, do not secrete protective cytokines (64). The protective function of CD4CD8αα IELs is executed mainly by IL-10, which is known as a suppressive cytokine. Das et al. (60) revealed that IL-10 is not required for the phenotypic development of CD4CD8^+^ T cells but is essential for their function. However, there is a strong dispute that IL-10 is produced by CD8^+^ IELs, not CD4^+^ IELs. CD8^+^ IELs are also the main producers of TGF-β, which assists IL-10 in exerting its immunosuppressive function (65). CD4CD8αα IELs also produce IL-10 and TGF-β, which can suppress Th1 cell-induced intestinal inflammation in an IL-10-dependent fashion (66). CD4CD8αα IELs mediate robust inhibition of CD4^+^ T cell proliferation and DC maturation so that they inhibit effector T cell responses when the intestinal tract is experiencing inflammatory conditions (67).

Sarrabayrouse et al. (68) found that between IBD patients and healthy individuals, the number of CD4CD8αα IELs was significantly different. In IBD patients, there are few CD4CD8αα IELs distributed in the intestine epithelia. This finding suggests that CD4CD8αα IELs are similar to Treg cells that exert immunosuppressive function and play an important role in intestinal homeostasis and IBD prevention. However, when the intestinal bacteria translocate, CD4CD8αα IELs exert mainly another special cytotoxic function (69). In IBD patients, with change in the intestinal bacteria, the cytotoxicity of CD4CD8αα IELs is activated, which leads to secretion of a large number of proinflammation cytokines such as TNF-α, IL-15, and IFN-γ, and upregulation of CD107a expression, implying the pathogenic potential of cytotoxic CD4CD8αα IELs (23).

Although many studies have indicated that CD4CD8αα IELs play important roles in protecting against invading pathogens. These cells have also been implicated in the pathological progression of IBD (70). When the invasion of pathogens is intense, CD4CD8αα IELs exert cytotoxic effects that may jeopardize the integrity of the mucosal barrier, making it easy for pathogens to penetrate into the intestinal tract (71). Furthermore, the invasion of pathogens results in CD4CD8αα IELs producing excessive IFN-γ, which may enhance HLA-E expression and promote cytotoxic responses of CD4CD8αα IELs through the innate CD94-NKG2D pathway (7, 72). This process is likely to be a key mechanism involved in the pathological progression of IBD. In addition, it remains possible, under inflammatory conditions or during recognition of foreign antigens that are highly similar to self-antigens, that CD4CD8αα IELs may drive autoimmune pathology, or CD4CD8αα IELs produce excessive IL-15, which may trigger their auto reactive cytotoxicity, therefore jeopardizing the integrity of the mucosal barrier (7, 73). Collectively, on the one hand, CD4CD8αα IELs can protect the intestinal tract from invasion by pathogens; on the other hand, they are also involved and play an important role in the pathological process of IBD.

## Conclusion

IELs are known as peripheral T cells with marked specificity and heterogeneity. Much progress has been made in recent years toward understanding the development and differentiation of various groups of IELs. However, the reprogramming of CD4^+^ T cells into intestinal intraepithelial CD4CD8αα IELs has not been recognized. It is conceivable that other CD4^+^ CTLs arise in humans and mice under inflammatory conditions during viral infections or in association with autoimmunity (9). Although CD4CD8αα IELs may have beneficial roles in IBD, the precise mechanism and the relevance of their cytolytic phenotypes are not yet understood. In addition, CD4CD8αα IELs may play a detrimental role in the progression of IBD, which makes the prognosis of IBD increasingly worse. Therefore, there is an exquisite balance between preventing intestinal inflammation and destroying intestinal tissue. Insight into these processes will help us better understand the biology of this significant yet greatly neglected T cell population, provide new insights to combat infections, and lead to the development of therapies to treat inflammatory diseases.

## Author Contributions

CZ conceived and wrote the manuscript. YQ and HY contributed to the writing and revision of the manuscript.

### Conflict of Interest

The authors declare that the research was conducted in the absence of any commercial or financial relationships that could be construed as a potential conflict of interest.
